# Smouldering disease in paediatric-onset multiple sclerosis

**DOI:** 10.1016/j.ebiom.2025.105921

**Published:** 2025-09-11

**Authors:** Massimo Filippi, Monica Margoni, Brenda Banwell, Tanuja Chitnis, Russell Dale, Giulia Fadda, Yael Hacohen, Lauren B. Krupp, Paolo Preziosa, E. Ann Yeh, Emmanuelle Waubant, Maria A. Rocca

**Affiliations:** a Neuroimaging Research Unit, Division of Neuroscience, IRCCS San Raffaele Scientific Institute, Milan, Italy; b Neurology Unit, IRCCS San Raffaele Scientific Institute, Milan, Italy; c Neurorehabilitation Unit, IRCCS San Raffaele Scientific Institute, Milan, Italy; d Vita-Salute San Raffaele University, Milan, Italy; e Neurophysiology Service, IRCCS San Raffaele Scientific Institute, Milan, Italy; f Department of Pediatrics, Johns Hopkins University, Baltimore, MD, USA; g Department of Paediatric Neurology, Massachusetts General Hospital, Harvard Medical School, Boston, MA, USA; h Kids Neuroscience Centre, Children's Hospital at Westmead Clinical School, University of Sydney, Sydney, NSW, 2145, Australia; i Department of Medicine, University of Ottawa, Ottawa Hospital Research Institute, Ottawa, ON, Canada; j Department of Neurology, Great Ormond Street Hospital for Children, London, UK; k Department of Neuroinflammation, UCL Institute of Neurology, University College London, London, UK; l Department of Neurology, NYU Grossman School of Medicine, New York, NY, United States; m Division of Neurology, Department of Pediatrics, The Hospital for Sick Children, University of Toronto, ON, Canada; n Department of Neurology, University of California San Francisco, San Francisco, CA, USA

**Keywords:** Chronic active lesions, Confirmed disability accrual, Fluid biomarkers, Grey matter, MRI, Neurofilaments, Paediatric-onset multiple sclerosis, Progression independent of relapse activity, Repair, Smouldering disease, Spinal cord, Treatment

## Abstract

Smouldering disease in multiple sclerosis (MS) refers to chronic central nervous system processes that occur beyond acute inflammation, driving long-term disability. Although current therapies effectively reduce relapse rates and MRI lesions, many individuals experience progression independent of relapse activity. While clinical progression is uncommon during childhood or adolescence, growing evidence suggests that subclinical progressive disease biology is already active even in this young age group, warranting early intervention to preserve function. Conventional MRI, while critical for diagnosis, lacks sensitivity for subtle damage. Advanced MRI techniques, including detection of chronic active lesions, global and focal brain damage, hold promise for early identification. Fluid biomarkers, such as neurofilament light chain and glial fibrillary acidic protein, provide non-invasive measures of neuroaxonal injury and ongoing chronic inflammation. This review summarises the role of MRI and fluid biomarkers in detecting smouldering disease in paediatric-onset MS and their application in supporting therapeutic decision-making.

## Introduction

Smouldering disease in multiple sclerosis (MS) refers to chronic, diffuse neurodegenerative processes that drive gradual clinical worsening beyond acute inflammation.^1^ These mechanisms are difficult to detect despite effective suppression of focal inflammation with current therapies. Paediatric-onset MS (POMS) offers insight into early disease biology, as its clinical onset is closer to the biological onset of disease than adult-onset MS (AOMS). Unique age-related features, such as the impact of the disease on brain maturational processes, as well as heightened plasticity of the central nervous system (CNS) in response to accumulating structural damage, are important facets of POMS as compared to AOMS.^2^, ^3^, ^4^, ^5^ Advanced magnetic resonance imaging (MRI) techniques offer promise for early detection of smouldering disease. They can capture subtle abnormalities in brain and spinal cord tissue that cannot be visualised using conventional imaging techniques.^6^, ^7^, ^8^, ^9^, ^10^, ^11^, ^12^ MRI markers of pathological processes potentially underlying smouldering disease are detectable in POMS. Serum and cerebrospinal fluid (CSF) biomarkers may reflect underlying neuroinflammation and axonal damage, supporting their role in identifying smouldering disease and guiding early intervention strategies.^13^, ^14^, ^15^, ^16^, ^17^

In this narrative review, we (1) summarise the immunopathology of smouldering disease and its clinical expression in POMS, (2) describe emerging imaging and body fluid markers used to identify smouldering disease, and (3) report the potential utility for neuroimaging and body fluid markers to monitor treatment effects.

## Search strategy and selection criteria

References for this narrative review were identified through searches of PubMed with the search terms “adult-onset multiple sclerosis”, “chronic active lesions”, “confirmed disability accrual”, “fluid biomarkers”, “grey matter”, “glial fibrillary acidic protein”, “MRI”, “neurofilaments”, “paediatric-onset multiple sclerosis”, “progression independent of relapse activity”, “repair”, “smouldering disease”, “spinal cord”, “thalamus”, “treatment”, “white matter” from 1990 until April, 2025. Each co-author performed a targeted literature search on the topic of their respective contribution, selecting the most relevant, recent, and innovative studies to support the scientific discussion. Articles were also identified through searches of the authors’ own files. Only papers published in English were reviewed. As this was not a systematic review, the number of records retrieved and a detailed screening process are not reported. The final reference list was generated on the basis of originality and relevance to the broad scope of this Review.

## Immunopathology of smouldering disease

Smouldering MS pathology is thought to result from widespread inflammatory and degenerative processes, including anterograde and retrograde axonal degeneration, impaired neuronal metabolism and mitochondrial dysfunction, oxidative stress, iron accumulation, glutamate excitotoxicity, coupled with the failure of reparative/compensatory mechanisms such as remyelination and neural plasticity.^1^ Our understanding of smouldering pathology is largely based on pathological studies performed in AOMS since, to date, no pathological studies have specifically characterised smouldering mechanisms in POMS. Nonetheless, these pathological processes are believed to underlie smouldering disease across the age span, and they may translate at least partially to POMS, even though with possible differences due to CNS maturation and immune responses. Increasing evidence suggests that CNS-intrinsic biological processes play a central role even in the early stages of MS and are considered distinct from the mechanisms underlying MS relapsing biology.

Several key processes may drive smouldering disease in MS. These include microglial activation around chronic active lesions (CALs) with B and T cell interactions, B cell activation associated with cortical demyelination, astrocyte-driven chronic inflammation, and intrinsic neuronal metabolic deficits.^1^ A network of glial, immune, and neural cells likely sustains the pathology of smouldering disease, reflected by the cellular composition of CALs behind an intact blood–brain barrier, containing activated microglia, astrocytes, oligodendrocytes, and lymphocytes.^9^ Diffuse microglial activation and oxidative injury in the normal appearing (NA) white matter (WM) correlate with axonal damage.^18^ Although microglia can be homoeostatic, they mainly promote harmful processes such as demyelination, aberrant synaptic pruning, and excessive phagocytosis.^1^ B and T cells in CNS and leptomeningeal chronic inflammation are also key drivers of smouldering disease. In the meninges, B cells can form tertiary lymphoid structures associated with subpial cortical demyelination.^19^ Even though CD8 T cells outnumber other T cells, Th17 cells have been proposed to be a potentially important cell type in smouldering MS, although targeting Th17 cells did not yield therapeutic benefits on the cumulative number of combined unique active lesions observed on brain MRI in a phase 2 trial.^20^ Focal grey matter (GM) damage, associated with meningeal inflammation, follows a surface-in gradient, causing severe demyelination and atrophy.^21^ Moreover, astrocytes activated by immune cells and microglia shift into neurotoxic states, further impairing neuronal metabolism, with mitochondrial dysfunction seen as a key driver of neurodegeneration.^22^ Due to the very limited pathological tissues (biopsies) or autopsy data in POMS, it is not possible to evaluate whether age at MS onset in childhood leads to differences in the pathological features of smouldering disease.

## Clinical features

### Disability worsening and progression independent of relapse activity

**Fig. 1:**
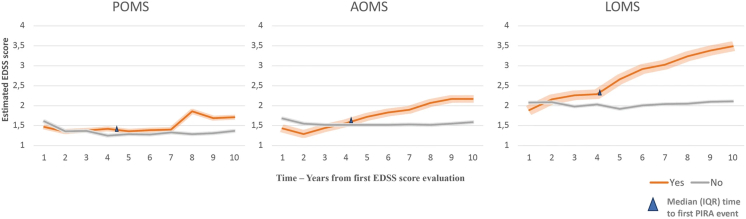
**Disability trajectories stratified by PIRA status in POMS, AOMS and LOMS**. In groups with AOMS and LOMS, individuals with PIRA (orange line) showed a significantly (p < 0.0001) steeper increase in EDSS scores than those without PIRA (grey line), and this was evident from the first year after the occurrence of PIRA. In the group with POMS, individuals who presented a PIRA event (orange line) had also a significantly (p < 0.0001) steeper increase in EDSS scores than those without PIRA (grey line), but, unlike AOMS and LOMS, the two disability trajectories began to diverge later, two years after the first PIRA event. “Yes” and “No” refer to the presence or absence of PIRA. Reproduced from: Simone M, Lucisano G, Guerra T et al. Disability trajectories by progression independent of relapse activity status differ in paediatric, adult and late-onset multiple sclerosis. J Neurol 2024; 271 (10): 6782-90, an open access article under a Creative Commons Attribution 4.0 International Licence (To view a copy of this licence, visit http://creativecommons.org/licenses/by/4.0/). Abbreviations. AO, adult onset; EDSS, Expanded Disability Status Scale; IQR, interquartile range; LO, late onset; MS, multiple sclerosis; PIRA, progression independent of relapse activity; PO, paediatric-onset.

The consequences of POMS on neurological function reflect relapse-related tissue injury, repair processes including remyelination and ongoing tissue loss driven by progressive MS pathobiological CNS injury. In a natural history cohort, individuals with POMS (median age at onset = 14.5 years) had a 50% chance of developing an expanded disability status scale (EDSS) score ≥6 at a median of 28.9 years post-first attack, and a median of 28 years to enter secondary progressive MS.^23^ In this study, in which the majority of individuals with POMS were untreated or treated with low to moderate efficacy therapies, the number of relapses in the first two years was associated with worse motor outcomes. However, the risk of progressive disability independent of relapses (PIRA, defined as confirmed disability accrual, CDA, more than 90 days following or more than 30 days prior to a relapse) is not easily discerned from this data. A more recent cohort study of over 16,130 individuals with MS evaluated PIRA, comparing 1383 POMS (median age at onset = 15.8 years) to 14,113 AOMS and 634 late-onset MS.^24^ POMS had more active disease, as measured by relapses, and appeared to have similar PIRA risk (40% of 558 individuals with CDA) compared to AOMS (44% of 6258 individuals with CDA). When PIRA was considered as a function of biological age at assessment, only 1.3% of individuals experienced PIRA at age 20 years, while 9% experienced PIRA by age 30 years. The rate of PIRA nearly doubled in each decade thereafter. Exposure to disease-modifying agents and duration of treatment reduced the likelihood of PIRA. An extension of this work evaluated disability trajectories of 268 individuals with POMS and 3282 with AOMS, stratified by the presence of PIRA.^25^ The rate of increase in disability was steeper in AOMS compared to POMS. Those with PIRA experienced faster accrual of disability, with a greater impact of PIRA being evident in AOMS. However, the absolute EDSS scores for POMS in this study were less than 2 across the years of evaluation, consistent with low levels of neurologic impairment (Fig. 1).^25^ Also noteworthy is that 97% of individuals with POMS in this cohort were treated within a few months of first attack, the majority with moderate efficacy treatments, although 62% escalated to high efficacy treatment (HET) over the period of observation. Current cohorts with POMS, particularly those reported with less than 10 years of observation, have median EDSS scores that remain very low, indicative of a very low rate of accrual of physical impairment, even if they meet criteria for a PIRA event.

### Cognitive impairment

PIRA can occur at any age, but is much less common while under 18 years of age.^24^ Nonetheless, at the time of diagnosis, children and adolescents have already lost some degree of brain volume relative to their non-MS peers,^10^ findings consistent with the concept of ongoing neurodegeneration and smouldering inflammation.

Individuals with POMS neurologically recover from relapses with fewer neurologic deficits than AOMS.^26^ Further, cognitive screening tests performed on POMS participants from the recent therapeutic era show little difference from similarly aged individuals without MS^27^ and POMS individuals had similar academic performance as their peers without MS.^28^

The biggest concern for smouldering inflammation among POMS is the long-term outcome of cognitive function. Among POMS individuals who underwent serial cognitive testing, a substantial subset had major cognitive declines after 10 or more years, which had adverse consequences for occupational attainment.^29^ In larger samples, cognitive processing speed performance early in the disease course was similar among individuals with AOMS and POMS, but over time POMS showed greater impairments with increasing disease duration.^30^

Environmental factors, specifically the social and economic conditions in which individuals are born and grown, have been found to be associated with cognitive reserve and structural brain outcomes in POMS.^31^ Among 138 POMS with a median age at onset of 15.5 years, indicators of socioeconomic hardship (e.g., public vs. commercial health insurance, having parents with low educational levels, or growing up in areas of neighbourhood deprivation) were associated with greater T2-hyperintense and T1-hypointense WM lesional volumes.^31^ Specifically, childhood neighbourhood social vulnerability index was the strongest individual predictor of total WM lesion (β = 4.63, p = 0.002) and T1-hypointense lesion volume (β = 2.91, p = 0.003). In the models incorporating all childhood social determinants of health variables, public health insurance was the strongest predictor of total lesion (β = 2.48, p = 0.01) and T1-hypointense lesion volume (β = 1.50, p = 0.02), attenuating the effect of neighbourhood social vulnerability index. Consistent with the critical role of cognitive reserve in predicting outcomes, lower baseline intelligence quotient is linked to greater GM volume loss and subsequent cognitive decline in POMS.^10^

Prompt diagnosis and initiation of HET prior to accumulation of neurologic impairment associates with a decreased likelihood of transition to a progressive course at 5 years.^32^ Also of therapeutic importance is implementing cognitive rehabilitation strategies for POMS.^33^

## Imaging markers

### WM lesions (slowly expanding lesions and paramagnetic rim lesions)

**Fig. 2:**
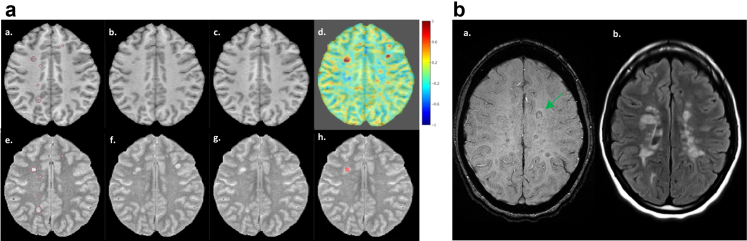
**SELs and PRLs in POMS**. a. Example of SEL detection as a region within an initial T2 lesion mask measuring at least 30 mm^3^ that shows radial and gradual expansion on serial T1-and T2-weighted MRI scans over three years in an individual with POMS (panels a–c for T1-and e–g for T2-weighted images). Volume changes are quantified at the voxel level through Jacobian determinant (panel d, where red voxels indicate expansion and blue voxels contraction). The boundaries of an identified SEL are marked in panel h. b. Detection of a PRL on axial susceptibility weighted images (a, green arrow) corresponding to an area of hyperintensity on T2 fluid-attenuated inversion recovery sequences in an individual with POMS (b). Only a minority of T2 hyperintense lesions show a paramagnetic rim on susceptibility weighted images. MRI images are obtained from the Pediatric Demyelinating Disease Study.^6^ Abbreviations: POMS, paediatric-onset multiple sclerosis; PRL, paramagnetic rim lesion; SEL, slowly expanding lesion.

**Fig. 3:**
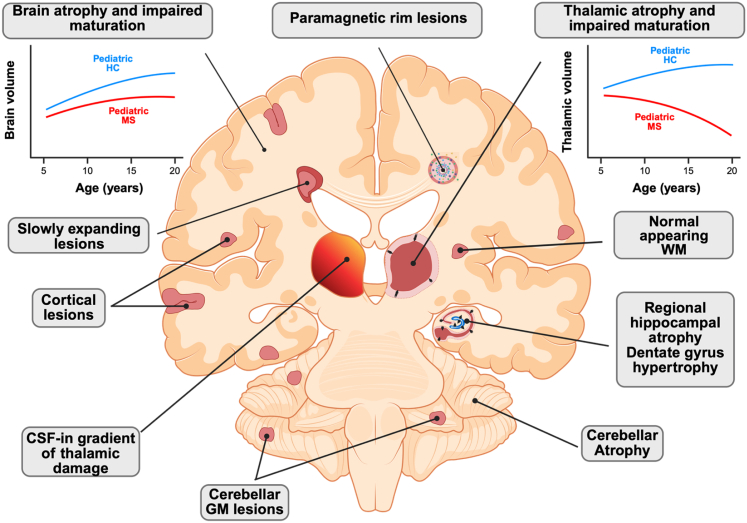
**Schematic overview of WM and GM pathological processes occurring in POMS**. Impaired brain and thalamic maturation have been observed in individuals with POMS, highlighting reduced age-related growth trajectories in these individuals compared to HC. Other features include cortical and cerebellar GM lesions, slowly expanding lesions, and paramagnetic rim lesions alongside microstructural changes in the normal-appearing WM. Additional findings involve hippocampal volume abnormalities, characterised by regional atrophy and dentate gyrus hypertrophy, and a CSF-in gradient of thalamic damage. Created with https://biorender.com. Abbreviations. CSF, cerebrospinal fluid; GM, grey matter; HC, healthy controls; POMS, paediatric-onset multiple sclerosis; WM, white matter.

The smouldering damage in MS manifests in multiple forms potentially arising from distinct sources, one of which is the downstream effects of acute inflammation. While most acute WM lesions evolve into inactive lesions, characterised by resolution of inflammation and demyelinating processes, others develop into CALs, marked by persistence of inflammation in the form of a rim of activated myeloid cells at the lesion edge, without major BBB breakdown.^34^ These latter lesions, often referred to as slowly expanding lesions (SELs), are believed to gradually enlarge over time, leading to progressive myelin and axonal damage. The detection of SELs relies on serial imaging acquisition over the course of several years, as WM lesions expand slowly. The defining imaging criteria for SELs include both gradual and radial expansion of pre-existing T2 lesions (Fig. 2a, Fig. 3), distinguishing them from the “new or enlarging lesions” detectable on conventional MRI, which encompass both new acute inflammatory activity near an existing lesion and chronic expansion.^34^ Although SELs are reported more frequently in the progressive phase of MS, recent studies on 19 and 40 children and adolescents with MS with a median age at MRI of 14.3 and 15.6 years identified the presence of SELs in 16 (84%)^6^ and 34 participants (85%),^35^ supporting the underlying progressive biology as an early feature of MS pathology.

CALs can be detected on MRI also due to the presence of iron-laden activated microglia/macrophages at the lesion edge, which are visible as a paramagnetic rim using susceptibility weighted MRI techniques (PRL) (Fig. 2b and 3).^36^ PRLs only partially overlap with SELs and might therefore be expression of distinct pathological aspects or phases of CALs.^34^ Recent paediatric studies, with cohort sizes between 10 and 13 participants (median ages at MRI ranging from 15.0 to 16.4 years), identified PRLs in 71–90% MS cases,^7^^,^^37^, ^38^, ^39^, ^40^ where they accounted for less of 5% of all WM lesions. Thus, despite being present in most individuals with MS, PRLs constitute only a minor fraction of WM lesions detected at any timepoint, a distinction that appears even more pronounced in POMS compared to AOMS.^7^

Both SELs and PRLs have high specificity for MS and are associated with more severe disease course,^6^^,^^38^ with significant diagnostic and prognostic implications. It must be acknowledged that PRLs and SELs are difficult to correlate with other markers of disability, such as EDSS score and brain volumes, due to the intrinsic repair/remyelinating capacity, which results in low clinical disability, and the ongoing brain maturational processes occurring in POMS. Despite these limitations, compared to SELs, PRLs have the advantage of being detectable at a single timepoint, enhancing their practical applicability as prognostic biomarker. The detection of PRLs will be incorporated in the diagnostic algorithm of the 2025 revision of the MS diagnostic criteria, and their identification among individuals with POMS supports the applicability of this neuroimaging biomarker across the age span.^40^

### Normal-appearing white matter (NAWM)

Abnormalities in the NAWM may contribute to clinical worsening independent of new inflammatory attacks in AOMS. Likewise, changes in WM microstructure precede GM atrophy and disability in POMS.^5^

Injury to NAWM in POMS can be demonstrated by using techniques such as diffusion tensor imaging (DTI), which evaluates properties of the diffusion of water through tissues. In the paediatric population, diffusion becomes increasingly restricted through time, a direct consequence of the expected development of myelin fibre bundles. Use of diffusion metrics, including mean diffusivity (MD) and fractional anisotropy (FA), can therefore demonstrate microstructural damage in NAWM or abnormalities in healthy myelin-related development.^41^ Other higher order diffusion methods such as neurite orientation dispersion and density imaging (NODDI)^42^ and fixel-based analysis (FBA),^43^ offer specificity with regards to neurite orientation and fibre density and therefore may be more sensitive to identifying axonal loss.

Cross-sectional work has consistently demonstrated widespread abnormalities in NAWM in POMS (Fig. 3). Specifically, decreases in FA and increase in mean, radial and axial diffusivity were demonstrated in cohorts of 10, 14 and 33 paediatric MS (median ages at MRI ranging from 15.1 to 16.6 years) compared to healthy children,^44^, ^45^, ^46^ with associations of worse outcomes with longer disease duration noted in one study.^44^ Specific areas of FA decrease have been seen in the splenium of the corpus callosum, right temporal, and right and left parietal regions as well as the occipital region. With regards to fibre density, children with MS (n = 18), but not myelin oligodendrocyte glycoprotein antibody-associated disease (n = 14) compared to healthy children (n = 26) had decreased fibre density and fibre bundle cross section in multiple areas, similar to those noted above, but also specifically in the corticospinal tracts and inferior longitudinal fasiculus.^43^ Similar results have been reported in a cohort of early-onset paediatric MS (onset before 12 years of age), in whom the main WM tracts exhibited different susceptibility to MS-related pathology, potentially reflecting age-dependent differences in the myelin and axonal characteristics of developing WM pathways.^47^

Using NODDI, neurite density has been found to be low in the corpus callosum and posterior thalamic radiation, with correlations between higher NAWM density and lesion volume in the cerebellar peduncle and corticospinal tract.^42^ NAWM changes are progressive as demonstrated in a longitudinal assessment (POMS = 52, healthy children = 80 and monophasic demyelination = 79). Individuals with POMS demonstrated progressive decreases in FA and increases in MD through time, suggesting both poor WM development and progressive loss of tissue integrity.^8^

### Focal and diffuse GM damage

Focal cortical lesions (CLs) are present in POMS, but less frequently than in AOMS (Fig. 3).^9^^,^^48^ A study using double inversion recovery (DIR) imaging reported CLs in 8% of individuals with POMS (median age at MRI = 15.5 years) vs. 66% of individuals with AOMS,^9^ with subsequent studies confirming low prevalence rates between 12 and 34%.^48^^,^^49^ Over time, CL accumulation occurs at similar rates regardless of age at onset (mean new CLs [standard deviation]: POMS = 1.5 [1.3]; AOMS = 1.1 [1.5]).^48^ In POMS, CLs are predominantly located at the GM–WM interface, a region where myelin proliferation persists through childhood and adolescence.^9^ Cerebellar GM lesions, detected in 93% of individuals with POMS at disease onset (mean age at MRI = 14.9 years) using phase-sensitive inversion recovery (PSIR) imaging (Fig. 3),^50^ are common. Despite their presence, GM lesions in POMS appear to have limited short-term clinical impact. The longer-term consequences of GM lesions in POMS are less clear.

POMS is also characterised by diffuse GM abnormalities, including microstructural alterations and atrophy in cortex, deep GM nuclei, hippocampus, and cerebellum.^5^^,^^10^^,^^48^^,^^51^, ^52^, ^53^, ^54^, ^55^ A key finding is the failure of normal age-expected GM maturation (Fig. 3).^10^^,^^51^ Individuals with POMS seem to deviate from expected brain growth trajectories, suggesting an impaired GM maturation and early GM volume loss.^10^^,^^51^ Interestingly, one study showed that individuals with POMS exhibit smaller brain and thalamic volumes compared to healthy peers, regardless of whether disease onset occurs before or after the age of 11.^51^ Moreover, the extent of GM volume abnormalities correlates with overall T2-hyperintense WM lesion volume, supporting the hypothesis that MS not only drives neurodegeneration secondary to inflammatory demyelination but also disrupts brain development when the disease occurs during critical periods of brain development. The thalamus seems particularly vulnerable to damage, showing early microstructural abnormalities and volume loss.^10^^,^^52^^,^^53^ This damage is at least partly due to disrupted thalamic connections caused by focal WM lesions, as indicated by strong associations with T2-hyperintense WM lesion volume.^52^ Additionally, recent studies have revealed a “surface-in gradient” of thalamic abnormalities, with the most severe damage near the CSF interface, suggesting the role of CSF-diffusible inflammatory or cytotoxic factors and of the choroid plexus in the onset and propagation of damage (Fig. 3).^52^^,^^53^^,^^56^ An association between thalamic microstructural damage and glymphatic system dysfunction (i.e., lower diffusion tensor image analysis along the perivascular space index values compared to healthy children) has also been recently observed in individuals with POMS (median age at MRI = 15.5 years).^57^ Additional GM volume abnormalities in POMS include bilateral radial atrophy of hippocampal body and head, with simultaneous radial hypertrophy of the cerebellar dentate gyrus (Fig. 3).^55^ These regional hippocampal abnormalities correlate with brain T2-hyperinense WM lesion volume and cognitive performance, suggesting that hippocampal atrophy may contribute to cognitive impairment in POMS, whereas dentate hypertrophy may represent a compensatory response.^55^ Cerebellar volume loss and posterior fossa lesions have been linked to impaired processing speed (Fig. 3).^54^

### Spinal cord damage

MRI studies have shown that spinal cord lesions in POMS are frequent, mainly localised at the cervical region and are typically focal and partial in cross-sectional involvement.^58^ Longitudinally extensive lesions have rarely been found in POMS (5%).^58^ The association of spinal cord lesions with disability accumulation has been assessed in a study of 125 individuals with POMS at their first demyelinating event, in which the number of baseline cervical cord lesions was found to be a moderate predictor (β = 0.22, p = 0.05) of higher EDSS 9 years after the baseline scan.^59^

Recent studies have also indicated that while microstructural abnormalities and global spinal cord volume in POMS may not differ significantly from healthy children,^11^^,^^12^^,^^60^ individuals with cervical cord lesions can exhibit voxel-wise GM abnormalities. One study investigating 38 individuals with paediatric MS with a median age at MRI of 17.4 years showed voxel-wise clusters of increased GM volume co-localising with cervical lesions,^11^ suggesting focal inflammation and oedema as predominant processes in the first phases of the disease. These findings mirror adult data, which shows preserved spinal cord volumes in the first phases of disease with subsequent development of atrophy with longer disease duration.^61^

A 12-year longitudinal study showed that individuals with relapsing-remitting AOMS who converted to secondary progressive MS exhibited faster spinal cord atrophy (−2.19% vs. −0.88% per year) beginning at least four years prior to conversion, highlighting the predictive value of spinal cord monitoring for early detection of smouldering disease.^62^ Longitudinal studies are needed to determine the prognostic relevance of spinal cord atrophy and the potential repair mechanisms for the spinal cord in paediatric cohorts.

### Tissue repair and plasticity

**Fig. 4:**
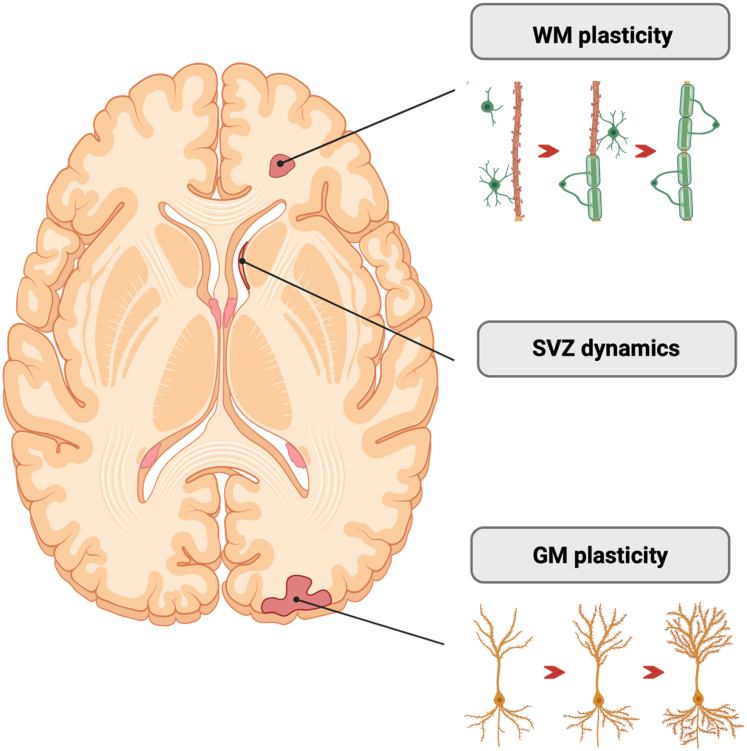
**Schematic overview of different mechanisms of tissue repair and plasticity occurring in POMS**. WM plasticity, including remyelination and axonal reorganisation, plays a central role in preserving network integrity in individuals with POMS. The SVZ contributes to regenerative processes through neurogenesis and glial cell differentiation. Additionally, GM plasticity—particularly dendritic remodelling and synaptic adaptation—may support cognitive reserve and functional compensation. Created with https://biorender.com. Abbreviations. CNS, central nervous system; GM, grey matter; POMS, paediatric-onset multiple sclerosis; SVZ, subventricular zone; WM, white matter.

The delayed accrual of motor disability in POMS, at least in the early years after disease onset, despite significantly more inflammatory activity compared to AOMS, may be attributed to more efficient repair mechanisms in POMS, to greater cellular reserve including mitochondrial function, and potentially to the reduced extent of spinal cord involvement, as well as to the absence of comorbid diseases that can deleteriously impact function. Consistent with the delayed accrual of motor impairment in POMS is the better recovery from clinical relapses^26^ and a slightly greater remyelinating capacity in acute focal lesions associated with younger age, as suggested by the higher amount of magnetisation transfer ratio recovery following lesion formation (Fig. 4).^4^ Recently, microstructural abnormalities in the subventricular zone, which harbours multipotent neural stem cells and progenitor cells, have been described in children with MS (median age at MRI = 14.6 years), but not in adults with POMS. These changes were associated with brain structural damage but not with clinical impairment.^2^

The CNS of children is also more “plastic” than that of adults.^63^ Structural and functional brain plasticity, especially in younger individuals with MS, may compensate for the progressive accumulation of MS-related structural damage. Activations of specific brain regions on functional MRI and distributed patterns of resting-state functional connectivity abnormalities in large-scale brain networks, compared with paediatric healthy controls and AOMS, have been suggested as possible adaptive compensatory mechanisms that preserve function despite the presence of extensive brain injury (Fig. 4).^3^

The enhanced repair capacity associated with POMS ultimately fails over time, with ageing and disease-related processes.^5^ Early in the disease, individuals with AOMS exhibit greater WM and GM damage than those with POMS. However, damage progresses faster in POMS, ultimately leading to more severe brain injury over longer disease durations.^5^ WM integrity abnormalities become more pronounced in POMS after 14 years of disease, whereas GM atrophy surpasses that of AOMS after 20 years. These findings suggest that while the younger brain initially compensates for MS-related damage, this ability diminishes over time, leading to progressive neurodegeneration, disability accumulation and cognitive deficits at a younger age.

## Body fluid biomarkers

Most available studies in paediatric populations primarily examine the relationship between biomarkers and clinical or imaging disease activity. Direct evidence linking these biomarkers to smouldering pathology or PIRA is currently lacking.

Oligoclonal bands and the kappa free light chain index (k-index) are well-established CSF biomarkers routinely used in the diagnostic work-up of POMS, reflecting intrathecal B-cell activation and immunoglobulin synthesis.^64^^,^^65^ However, to date, there is no evidence supporting their prognostic value specifically in relation to PIRA in POMS. Similar to AOMS, neurofilament light chain (NfL) level in serum and CSF is an established marker of concurrent disease activity (i.e., recent/ongoing clinical relapse, new T2 lesions, or enhancing MRI lesions) in POMS.^13^, ^14^, ^15^, ^16^^,^^66^ In the same way, serum glial fibrillary acidic protein (GFAP) reflects ongoing CNS injury, more specifically involving astrocytes.^16^^,^^17^ Although studies in individuals with POMS have not been performed yet, some evidence showed that in individuals with AOMS, including younger subjects, higher baseline sGFAP levels may be predictive of subsequent PIRA development.^67^ This suggests that GFAP may capture a different component of disease biology, namely, astrocytic dysfunction or glial responses, in addition to axonal damage reflected by NfL, and possibly associated to chronic inflammation and neurodegeneration. Other biomarkers, such as tryptophan metabolites and plasma lipid profiles, have been evaluated primarily in individuals with POMS and should be considered as preliminary markers of disease activity in POMS^17^^,^^68^, ^69^, ^70^ unless also reported in adult MS. While these research markers may reflect systemic metabolic or immune alterations, their relevance for smouldering disease or PIRA remains to be clarified, as is their place for clinical use in paediatric populations.

Therefore, there is an urgent need for well-designed longitudinal studies investigating the association of serum and CSF biomarkers in smouldering disease and their predictive value in POMS. These studies should ensure appropriate normalisation of biomarker measurements to account for age-related variability and other potential confounders (i.e., body mass index), thereby improving the reliability and clinical applicability of these markers in paediatric populations.

## Therapeutic implications

Smouldering disease starts early in POMS and PIRA must be considered as a target in the therapeutic management of POMS. Moreover, as more sensitive biomarkers of PIRA are developed, we are likely to discover a higher incidence and better understanding of PIRA in POMS.^16^ It is unclear whether the current therapies commonly used for POMS reduce PIRA in this early-onset population.^71^ Although a recent study performed in a small group of individuals with POMS suggested that natalizumab may prevent PIRA over a mean follow-up period of 46 months,^72^ long-term data are needed to confirm these findings. A delayed DMT start is associated with an increased risk of disability accrual in individuals with POMS.^24^^,^^73^ Individuals starting on a DMT later than 2 years after onset had a 2.52-fold increased risk of reaching sustained EDSS 4 compared to those starting within 2 years of onset.^73^ The reduction of PIRA must be incorporated into the analysis of future POMS clinical trials. It is important to note that clinical trials typically have a two-year follow-up and assessing PIRA is particularly challenging when relying on the EDSS score. Therefore, identifying alternative markers of progressive disease, such as SELs and PRLs, is crucial in POMS. New therapies that more specifically target smouldering or trapped inflammation in MS are being developed including the Bruton's tyrosine kinase inhibitor class^74^ and other therapies targeting activated microglia. Because individuals with POMS have a high relapse rate and a high rate of relapse associated worsening,^75^ therapeutic strategies must effectively address relapses as well as PIRA in our youngest individuals.

## Conclusions

Individuals with POMS offer a unique lens to investigate smouldering disease processes contributing to disability accumulation. Insidious changes begin early and are often undetected by conventional MRI. Advanced imaging and fluid biomarkers hold promise for identifying early signs of smouldering disease and guide therapeutic strategies. Although early HETs appear to mitigate PIRA risk, dedicated paediatric trials and biomarker-driven studies, including individuals with early POMS, are needed. A combined approach on early detection, precision diagnostics, and targeted therapies offers a path forward to reduce long-term disability and optimise outcomes in POMS.

## Outstanding questions

• Can serum or cerebrospinal fluid biomarkers such as neurofilament light chain or glial fibrillary acidic protein associate with smouldering disease in children and adolescents with MS?
• What are the long-term implications of early smouldering pathology for cognitive and functional outcomes in individual with paediatric MS?
• Can paramagnetic rim lesions be measured in clinical practice, using appropriate MRI sequences?
• Research studies dedicated to evaluation of slowly expanding lesions over time are required to determine whether SELs inform on long-term outcomes.
• Which therapeutic strategies are most effective in halting smouldering progression in POMS?

## Contributors

MF, MM, MAR contributed to methodology, conceptualisation, visualisation, writing—original draft, and writing—review and editing. BB, TC, RD, GF, YH, LBK, PP, AY, EW contributed to writing—original draft, literature search, and writing—review and editing. All authors read and approved the final version of the manuscript.

## Declaration of interests

MF is Editor-in-Chief of the *Journal of Neurology*, Associate Editor of *Human Brain Mapping*, *Neurological Sciences*, and *Radiology*; received compensation for consulting services from Alexion, Almirall, Biogen, Merck, Novartis, Roche, Sanofi; speaking activities from Bayer, Biogen, Celgene, Chiesi Italia SpA, Eli Lilly, Genzyme, Janssen, Merck-Serono, Neopharmed Gentili, Novartis, Novo Nordisk, Roche, Sanofi, Takeda, and TEVA; participation in Advisory Boards for Alexion, Biogen, Bristol-Myers Squibb, Merck, Novartis, Roche, Sanofi, Sanofi-Aventis, Sanofi-Genzyme, Takeda; scientific direction of educational events for Biogen, Merck, Roche, Celgene, Bristol-Myers Squibb, Lilly, Novartis, Sanofi-Genzyme; he receives research support from Biogen Idec, Merck-Serono, Novartis, Roche, the Italian Ministry of Health, the Italian Ministry of University and Research, and Fondazione Italiana Sclerosi Multipla. MM reports grants and personal fees from Sanofi Genzyme, Merck Serono, Roche, Biogen, Amgen and Novartis. BB provides nonremunerated advice on clinical trial design to Novartis, Biogen, and Teva Neuroscience. BB is funded by the National Multiple Sclerosis Society, National Institutes of Health, and Multiple Sclerosis Society of Canada. TC has received compensation for consulting from Bristol Myers Squibb, Cabaletta Bio, Genentech∗, Janssen, Merck KGaA, MJH Life Sciences, Novartis Pharmaceuticals AG, Novartis Pharmaceuticals KK, Octave Bioscience, F. Hoffmann-La Roche Ltd, Sanofi, Siemens, and UCB Biopharma SRL. Dr. Chitnis has received research support from the BrightFocus Foundation, Bristol Myers Squibb, Genentech, EMD Serono, I-Mab Biopharma, Massachusetts Life Sciences Center, National Institutes of Health, National MS Society, Novartis Pharmaceuticals, Octave Bioscience, Sanofi Genzyme, Tiziana Therapeutics, US Department of Defence, and Wesley Clover International. All activities and funding have occurred within the past 24 months (∗relationship has since ended) and disclosures do not conflict with the work being presented). RD participated in Data Safety Monitoring Board for Roche IDMC for paediatric MS ocrelizumab v fingolimod double blind double sham placebo controlled trial (2022-current). GF reports a relationship with Novartis Pharmaceuticals, Roche and Amgen Canada Inc that includes: consulting or advisory. GF reports a relationship with Brain Canada Foundation that includes: funding grants. GF has received Lecture Honorarium from AAN, CMSC and CAR, and travel support from Roche. YH reported grant from MS Society; honoraria for writing from American Academy of Neurology and for lectures from Cemcat Barcelona; and travel grant from Guthy Jackson Foundation. LBK has received research or programmatic funding or has received compensation for consulting, speaking, travel and meal allowances, or serving on DSMB committees from Ebix, Gerson Lehrman, WebMD, Novartis, Biogen, F. Hoffman/LaRoche, Bristol Myers Squibb, Celgene, Alexion, Amgen and EMD Serono. She has been compensated for providing consultative services for the legal firms KBR LLP, Segwick CMS, Faggiano and Associates Risk Management and MCIC Vermont. She also receives royalties for use of the Fatigue Severity Scale by various biopharmaceutical entities. All activities and funding have occurred within the past 36 months. PP received speaker honoraria from Roche, Biogen, Novartis, Merck, Bristol Myers Squibb, Genzyme, Horizon and Sanofi, he has received research support from Italian Ministry of Health and Fondazione Italiana Sclerosi Multipla. EAY has received research support in the last 3 years from the National Multiple Sclerosis Society, Canadian Institutes of Health Research, National Institutes of Health, Ontario Institute of Regenerative Medicine, Stem Cell Network, SickKids Foundation, Peterson Foundation, Multiple Sclerosis Society of Canada, Guthy Jackson Foundation, OMS Life, Canada's Drug Agency, Garry Hurvitz Chair in Neurology and the Multiple Sclerosis Scientific Research Foundation. She has served on scientific advisory boards for Biogen, Alexion, and Hoffman-LaRoche. DSMB: WCG. Co-chief Editor: MS and Related Disorders. Speaker/other Honoraria/Support for Travel: SOPNIA Chile, University of Chile, ECTRIMS, ACTRIMS, Johns Hopkins University, New Brunswick Neurological Society, American Academy of Neurology, Consortium of MS Centres, University of Ottawa, Canadian Institutes of Health Research, Michael Smith Health Research Organisation, Medlink. Clinical trials: Alexion, Novartis, Hoffman-LaRoche. Governing Council/Steering Committee: Stem Cell Network, Rare Kids CAN, Cantrain. EW is funded by the National Mutliple Sclerosis Society, the National Institutes of Health, PCORI and the Race to Erase MS. EW volunteered on Data Safety Monitoring Board or Advisory Board for BMS DSMB and Roche; her institution received/receives grants from NIH, DoD, Race to Erase MS, NMSS and Amgen. MAR received consulting fees from Biogen, Bristol Myers Squibb, Roche; and speaker honoraria from Alexion, Biogen, Bristol Myers Squibb, Celgene, Horizon Therapeutics Italy, Merck Serono SpA, Mitsubishi-Tanabe Pharma, Neuraxpharm, Novartis, Roche, Sandoz, and Sanofi. She receives research support from the MS Society of Canada, the Italian Ministry of Health, the Italian Ministry of University and Research, and Fondazione Italiana Sclerosi Multipla. She is Associate Editor for *Multiple Sclerosis and Related Disorders*; and Associate Co-Editor for Europe and Africa for *Multiple Sclerosis Journal*.
